# Is Sleep Associated with the S-Klotho Anti-Aging Protein in Sedentary Middle-Aged Adults? The FIT-AGEING Study

**DOI:** 10.3390/antiox9080738

**Published:** 2020-08-12

**Authors:** Sol Mochón-Benguigui, Almudena Carneiro-Barrera, Manuel J. Castillo, Francisco J. Amaro-Gahete

**Affiliations:** 1 EFFECTS-262 Research Group, Department of Medical Physiology, School of Medicine, University of Granada, 18016 Granada, Spain; mcgarzon@ugr.es; 2 Sleep and Health Promotion Laboratory, Mind, Brain and Behaviour Research Centre (CIMCYC), University of Granada, 18011 Granada, Spain; acarneiro@ugr.es

**Keywords:** successful ageing, inflammation, oxidative stress, accelerometry

## Abstract

Sleep and Klotho have both been closely related to the ageing process, both playing a substantial role in the endocrine and immune systems and, thereby, in oxidative stress and chronic inflammation. However, there are no studies elucidating the relationship between sleep and Klotho. Therefore, this study investigated the association of sleep quantity and quality with the shed form of the α-Klotho gene (S-Klotho plasma levels) in sedentary middle-aged adults. A total of 74 volunteers (52.7% women; aged 53.7 ± 5.1) were recruited for the present study. Objective sleep quality parameters (total sleep time (TST), wake after sleep onset (WASO), and sleep efficiency (SE)) were determined using a wrist-worn accelerometer over seven consecutive days, and the subjective sleep quality was assessed by the Pittsburgh Sleep Quality Index (PSQI; higher scores indicate worse sleep quality). The S-Klotho plasma levels were measured in the ethylenediaminetetraacetic acid plasma using a solid-phase sandwich enzyme-linked immunosorbent assay. Objective sleep parameters were associated with the S-Klotho plasma levels only after including the age, fat mass percentage, and lean mass index as covariates. A direct relationship was observed between the subjective sleep quality (inverse of PSQI scores) and the S-Klotho plasma levels in sedentary middle-aged adults. Improving sleep quantity and quality could be considered an anti-aging therapeutic approach for the prevention, slowing, and even reversal of the physiological decline and degenerative pathologies that are certainly related to the aging process.

## 1. Introduction

The increasing population ageing occurring worldwide, with the subsequent upsurge in vulnerability to morbidity and age-related diseases among adults, has certainly become one of the most significant global clinical and economic burdens for health systems and all aspects of society. According to the Global Burden of Disease Study 2017 [1], 51.3% of all burdens among adults were identified as age-related diseases, mostly including non-communicable diseases such as neoplasms, cardiovascular diseases, chronic respiratory diseases, diabetes and kidney diseases, digestive diseases, and neurological disorders among others [2]. In response to this remarkable demographic transition, the World Health Organization developed a global strategy and action plan on ageing and health in 2017 with goals and strategic objectives focusing on health system alignment to the needs of the older population and the enhancement of measurement, monitoring, and research to support healthy ageing [3,4].

In this context, the elucidation of aging mechanisms by advances in medicine and research has led to the emergence of anti-aging medicine, a growing field of research and clinical practice focused on the prevention, slowing, and even reversal of the physiological decline and degenerative pathologies related to the aging process [5,6,7,8,9]. The approaches of anti-aging medicine include, among others, calorie restriction mimetics, hormonal replacement, and gut microbiota and vitamin D interventions [5]. In this field of research, sleep could be considered a substantial key element involved in the restoration and preservation of multiple physiological systems, including the endocrine function and metabolism, immune response, and general brain metabolism [10,11,12]. Indeed, sleep deprivation has been closely related to energy imbalance [13,14], adverse hormonal changes [15], gut microbiota alterations [16], and vitamin D deficiency [17], all involved in the mechanisms of action of anti-aging medicine interventions. Accordingly, gathered evidence has also widely shown that sleep disturbances certainly lead to a vast number of age-related diseases [18], including obesity [19,20], cardiovascular disease [21], type II diabetes mellitus [22,23], chronic kidney disease [24], and psychiatric disorders [25].

Similarly, the Klotho gene family has been established as an “aging-suppressor” factor that accelerates aging when disrupted and extends life span when overexpressed through a wide variety of mechanisms [26,27,28,29,30]. Specifically, evidence suggest that α-Klotho—a single-pass transmembrane glycoprotein encoded by the Klotho gene and mainly expressed in the kidneys and brain choroid plexus—modulates the insulin-like growth factor and Wnt signaling pathways; inhibits oxidative stress; and regulates the metabolism of phosphate, calcium, and vitamin D in humans [28,29]. Within the three identified α-Klotho protein types [31], its secreted (or soluble) form (S-Klotho)—expressed in the blood, plasma, urine, and cerebrospinal fluid—works as a circulating hormone with significant metabolic functions on different tissues and organs, including anti-inflammatory and anti-oxidative stress effects [29,32,33]. Thus, the S-Klotho identified in plasma levels, which has been robustly associated with the α-Klotho gene expression [34], may be a powerful biomarker of biological anti-aging and, in turn, a promising therapeutic target for the prevention of aged-related disorders.

Sleep and S-Klotho, therefore, share common underlying mechanisms and physiological pathways through which they are linked to the ageing process in adults, both playing a positive substantial role in the endocrine [32,35,36,37] and immune systems [38,39] and, thereby, in oxidative stress [33,40] and chronic inflammation [29,41,42]—The leading molecular mechanisms behind all age-related consequences [43,44,45,46]. Yet, the available evidence on the relationship between sleep and S-Klotho is remarkably limited. A previous study by Pákó et al. [47] found reduced levels of S-Klotho in patients with obstructive sleep apnoea, potentially enhancing the systemic inflammation and endothelial dysfunction associated with this sleep-related breathing disorder. Similarly, another empirical study also concluded that sleep deprivation had an adverse effect on S-Klotho responses to exercise testing in healthy adults [48]. Conversely, the results from a recent study on the role of S-Klotho as a potential biomarker of stress exposed that unsatisfactory sleep was positively related to increased S-Klotho levels, although it is worth mentioning that sleep was only subjectively measured [49].

Hence, our study was aimed at elucidating the potential association of objective and subjective sleep duration and quality—including total sleep time (TST), sleep efficiency (SE), wake after sleep onset (WASO), and other subjective sleep parameters—with the S-Klotho plasma levels in sedentary middle-aged adults. In accordance with most but not all available evidence, we hypothesized that better sleep quantity and quality would be significantly associated with the increased plasma levels of S-Klotho.

## 2. Materials and Methods

### 2.1. Study Protocol and Participants

A total of 74 sedentary healthy middle-aged volunteers (52.7% women, 53.7 ± 5.1 years old, 26.7 ± 3.8 kg/m^2^) were recruited for the FIT-AGEING study [50], an exercise-based randomized controlled trial (clinicaltrial.gov: ID: NCT03334357), approved by the Human Research Ethics Committee of the “Junta de Andalucía” (0838-N-2017). A detailed explanation of the study methodology can be found elsewhere [50]. The study was in accordance with ethical principles of the Declaration of Helsinki. All the participants were given a full explanation of the study, completed a written consent form, and underwent a complete medical and physical examination prior to their enrolment in the study. The inclusion criteria were: (i) being aged from 40 to 65 years, (ii) having a body mass index (BMI) between 18.5 and 35 kg/m^2^, (iii) having a stable weight in the last three months (weight changes < 3 kg), (iv) being a non-smoker, and (v) being sedentary (i.e., self-reported <150 min of moderate-intensity aerobic physical activity throughout the week or <75 min of vigorous-intensity aerobic physical activity throughout the week). The exclusion criteria were: (i) having some acute or chronic illness, (ii) taking some medication, and (iii) being pregnant.

### 2.2. Measurements

#### 2.2.1. Anthropometry and Body Composition

Body weight and height were measured using an electronic scale and stadiometer (model 799, Electronic Column Scale, Hamburg, Germany). BMI was calculated as weight (kg)/height^2^ (m^2^) [51].

A dual-energy X-ray absorptiometry scanner (Hologic, Inc., Bedford, MA, USA) was used to determine the fat mass and lean mass. The fat mass index (FMI) and lean mass index (LMI) were calculated as fat mass (kg)/Height^2^ (m^2^), and lean mass (kg)/Height^2^ (m^2^), respectively.

#### 2.2.2. Sleep Quantity and Quality

The objective characteristics of sleep-wake patterns were assessed with a wrist-worn accelerometer (ActiGraph GT3X+, Pensacola, FL, USA) continuously 24 h a day for seven consecutive days [50]. The participants received detailed instructions to wear the accelerometer on the non-dominant wrist and to remove it during water activities. They were provided with a seven-day sleep diary to record bed-time, wake up time, and the time they removed the device each day. The accelerometer was initialized to store raw accelerations at a sampling frequency of 100 Hz [52]. The data were processed using ActiLife software (version 6.13.3, ActiGraph, Pensacola, FL, USA). The GT3X+ files were subsequently converted to 1 s epoch csv files containing x, y, and z vectors to facilitate raw data processing. These files were processed in R (version 3.1.2, https://www.cran.r-project.org/) using GGIR package (version 1.5-12, https://cran.r-project.org/web/packages/GGIR/). The signal processing included: (i) auto-calibration using local gravity as a reference [53], (ii) the detection of sustained abnormal high accelerations, (iii) the detection of non-wear time, (iv) the calculation of the Euclidean Norm Minus One (ENMO), (v) the calculation of waking and sleeping time by an automatized algorithm [54], and (vi) the imputation of abnormal high values and detected non-wear time. The variables analyzed from actigraphy recordings were WASO (the sum of wake times from sleep onset to the final awakening), TST (total amount of time spent in bed minus sleep onset latency), and SE (percentage of sleep time over the bedtime) [55]. Adherence was defined as ≥ 16 h/day of wear time for at least four of seven possible days of wear (including at least one weekend day).

The subjective sleep quantity and quality were measured by the Pittsburgh sleep quality index (PSQI) scale [56]. The PSQI contains 19 self-rated questions for scoring, combined into seven components, each of them with a range of 0–3 points: (i) subjective sleep quality, (ii) sleep latency, (iii) sleep duration, (iv) habitual sleep efficiency, (v) sleep disturbances, (vi) the use of sleeping medication, and (vii) daytime dysfunction. A global PSQI score is obtained by the sum of the seven components ranging from 0 to 21. Lower scores denote a healthier sleep quality, whereas a global score of more than five indicates poor sleep quality.

#### 2.2.3. S-Klotho Plasma Levels

Blood samples were collected from the antecubital vein applying standard techniques after overnight fasting. The samples were centrifuged and collected at the same time (8:30 a.m.–10 a.m.), processed in a controlled-temperature room (22 ± 0.5 °C), and kept in a −80 °C freezer (i.e., 6 months before the analysis). A solid-phase sandwich enzyme-linked immunosorbent assay (Demeditec, Kiel, Germany) was used to measure the S-Klotho plasma levels in the ethylenediaminetetraacetic acid plasma, which was previously validated by both the manufacturer (obtaining a sensitivity analysis of 6.15 pg/mL) and our own research group (obtaining intra- and inter-assay coefficients of variation which ranged from ~3% to ~10%). All the participants were requested to abstain from caffeine and/or drugs, to eat a standardized dinner before sampling, and to refrain from any physical activity of moderate (24 h before) and/or vigorous intensity (48 h before).

### 2.3. Statistical Analysis

The normal distribution of the variables was tested through the Shapiro–Wilk test and a visual check of histograms, Q-Q, and box plots. The descriptive parameters were reported as the mean and standard deviation. Independent sample T-tests were performed to determine sex differences.

Simple linear regression models were conducted to examine the association of sleep quality (TST, WASO, SE, and global PSQI score) with the S-Klotho plasma levels. Multiple linear regression models were also performed to test these associations after adjusting by age, fat mass percentage, FMI, and LMI.

Statistical Package for the Social Sciences (SPSS, version 23.0, IBM SPSS Statistics, IBM Corporation, Armonk, NY, USA) was used for the data analyses. *P* values less than or equal to 0.05 were considered statistically significant. GraphPad Prism 6 (GraphPad Software, San Diego, CA, USA) was used to create all the graphical presentations.

## 3. Results

### 3.1. Study Participants

The study participants’ characteristics can be found in Table 1. Significant differences between sex were observed in height, weight, BMI, fat mass percentage, LMI, TST, WASO, SE, subjective sleep quality (PSQI component), and habitual sleep efficiency (PSQI component) (all *p* < 0.033). A poor subjective sleep quality (global PSQI score > 5) was identified in 40.3% of the population.

### 3.2. Association between Objective Sleep Quantity and Quality and S-Klotho

**Figure 1:**
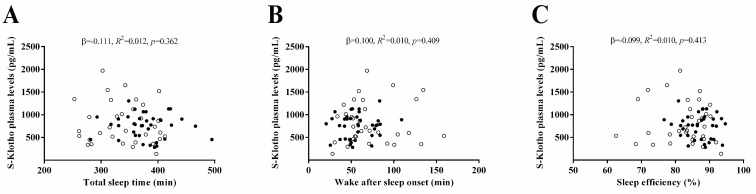
Association of objective sleep quantity and quality with the S-Klotho plasma levels in sedentary middle-aged adults. *β* (standardized regression coefficient), *R^2^*, and *p* from a simple linear regression analysis. Significant *p* values (<0.05) are highlighted in bold. Open circles represent men, close circles represent women, and the straight solid line represents the regression line. S-Klotho = Secreted Klotho.

Figure 1 shows the association of objective sleep quantity and quality with the S-Klotho plasma levels. TST, WASO, and SE were not associated with the S-Klotho plasma levels (all *p* > 0.05, Figure 1A–C).

Table 2 shows the relationship of objective sleep quantity and quality with the S-Klotho plasma levels adjusted by age, fat mass percentage, FMI, and LMI in the statistical models. An association of TST, WASO, and SE with S-Klotho appeared after including age, fat mass percentage, and LMI as covariates.

### 3.3. Association between Subjective Sleep Quantity and Quality and S-Klotho

**Figure 2:**
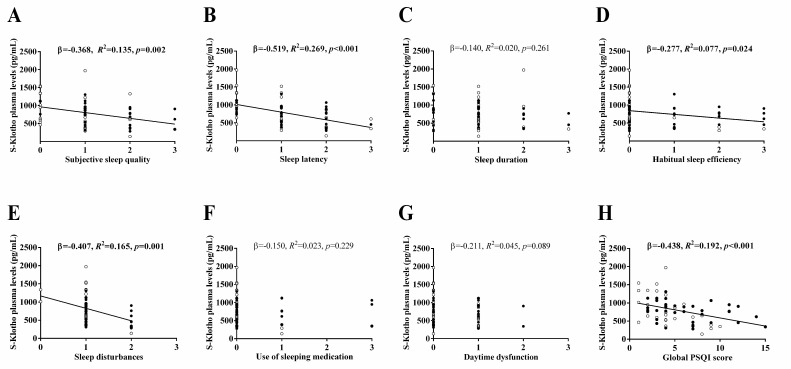
Association of subjective sleep quantity and quality (components and global PSQI score) with the S-Klotho plasma levels in sedentary middle-aged adults. *β* (standardized regression coefficient), *R^2^*, and *p* from a simple linear regression analysis. Significant *p* values (<0.05) are highlighted in bold. Open circles represent men, close circles represent women, and the straight solid line represents the regression line. S-Klotho = Secreted Klotho; PSQI = Pittsburgh Sleep Quality Index.

Figure 2 shows the association of subjective sleep quantity and quality (components and global PSQI score) with the S-Klotho plasma levels. An inverse relationship was observed between the global PSQI score and the S-Klotho plasma levels (*β* = −0.438, *R^2^* = 0.192, *p* < 0.001, Figure 2H), meaning that a higher subjective sleep quality was significantly related to increased S-Klotho plasma levels. Furthermore, our results showed the inverse associations of subjective sleep quality, sleep latency, habitual sleep efficiency, and sleep disturbance PSQI components with the S-Klotho plasma levels (*β* = −0.368, *R^2^* = 0.135, *p* = 0.002, Figure 2A; *β* = −0.519, *R^2^* = 0.269, *p* < 0.001, Figure 2B; *β* = −0.277, *R^2^* = 0.077, *p* = 0.024, Figure 2D; *β* = −0.407, *R^2^* = 0.165, *p* = 0.001, Figure 2E, respectively). Therefore, better sleep quality and efficiency, shorter sleep latency, and lower levels of sleep disturbances were all related to higher plasma levels of S-Klotho. We did not observe any significant associations between sleep duration, the use of sleeping medication, and daytime dysfunction with the S-Klotho plasma levels (all *p* > 0.05, Figure 2C,F,G).

Table 3 shows the relationship of subjective sleep quantity and quality (components and global PSQI score) with the S-Klotho plasma levels adjusted by age, fat mass percentage, FMI, and LMI in the statistical models. All of the above-mentioned findings persisted once age, fat mass percentage, FMI, and LMI were included in the statistical models. Furthermore, the associations between sleep duration, the use of sleeping medication, and daytime dysfunction with the S-Klotho plasma levels appeared after adjusting by age, fat mass percentage, and LMI.

## 4. Discussion

Our study sought to elucidate the relationship between sleep quantity and quality and the S-Klotho plasma levels in sedentary middle-aged adults. As we expected, the results from the current study indicated that objective sleep quantity and quality parameters (TST, WASO, and SE) were significantly associated with the S-Klotho plasma levels when age, fat mass percentage, and LMI were considered as covariates. Furthermore, we observed that better subjective sleep quality (measured by the global PSQI score) was related to higher levels of S-Klotho plasma levels in sedentary middle-aged adults. These results therefore have important clinical implications, as improving sleep quantity and quality could be considered a novel anti-aging therapeutic approach via increasing the S-klotho plasma levels.

Sleep quantity and quality represent a cornerstone in the maintenance of health and well-being, particularly during the senescence process and thus in the prevention of numerous degenerative chronic diseases [57,58,59,60,61,62]. Poor sleep quality and both short and long sleep duration have been previously associated with a lifespan reduction due to the associated deleterious effects on health and the higher risk of several diseases [18,63]. Sleep and S-Klotho have both been widely shown to be related to the ageing process [29,32,33,35,36,37,38,39,40,41,42], sharing underlying mechanisms and physiological pathways such as endocrine functions and metabolism, immune response and, consequently, oxidative stress and chronic inflammation. However, the relationship between these two aged-related factors still remains unclear. According to our results, better subjective sleep quality was positively related to enhanced levels of S-Klotho. A previous study by Nakanishi et al. [49], however, conversely found that subjective sleep dissatisfaction was related to higher levels of this anti-aging protein. This inverse association could be explained by a compensatory mechanism triggered to counteract the inflammatory stress-derived environment produced by sleep deprivation [49]. Nevertheless, as previously mentioned, it is also noteworthy that sleep in the study by Nakanishi et al. [49] was solely measured using one item on “relaxation from sleep”.

Regarding objective sleep and in accordance with previous findings [47,48], our results indicated that shorter sleep was related to reduced S-Klotho plasma levels after adjusting by fat mass percentage and LMI. These results are in accordance with previous findings [47,48], where sleep deprivation and obstructive sleep apnea—which causes short sleep and sleep fragmentation due to the repetitive collapse of the upper airway during sleep—were related to lower levels of the anti-aging protein. According to the evidence, the short sleep and chronic intermittent hypoxia caused by sleep apnea are both closely related to energy imbalance and obesity [13,14], adverse hormonal changes [15], gut microbiota alterations [16], vitamin D deficiency [17], and thus systemic inflammation and oxidative stress [40,41,42,47]. These physiological consequences, in turn, may lead to reduced S-Klotho levels, as it is known that factors such as obesity, vitamin D deficiency, and systemic inflammation suppress renal klotho synthesis [47,64,65]. Subsequently, a reduction in the S-Klotho expression may result in endothelial dysfunction, excessive aldosterone production, hypertension, renal structure damage, and functional decline, exacerbating therefore the increased systemic inflammation and oxidative stress found in sleep disturbances such as obstructive sleep apnea and/or sleep curtailment [47,66,67].

To the best of our knowledge, this is the first study describing the relationship between sleep quantity and quality and the S-Klotho plasma levels in healthy sedentary middle-aged adults. Our results therefore have robust clinical and research implications, supporting the association of better sleep quantity and quality with increased plasma levels of S-Klotho. Considering the increasingly high prevalence of sleep disturbances and its association with age-related disorders [68,69,70,71,72,73,74], promising anti-aging interventions should consider sleep as a modifiable factor for healthy aging. In this regard, the measurement of S-Klotho plasma levels could be used as a marker of a healthier and anti-aging sleep.

However, our study has some limitations that need to be addressed in future studies. Firstly, the cross-sectional study design used does not allow the identification of any causal association between the variables included, so well-designed longitudinal studies are needed to robustly analyze and establish causal relationships between sleep and S-Klotho. Secondly, the participants included in our sample were healthy sedentary middle-aged adults, such that these findings cannot be extrapolated to other individuals with different biological characteristics. Finally, although we used accelerometry as an objective tool to assess sleep quantity and quality, future studies should include polysomnography, which is the gold-standard method to appropriately assess not only sleep duration and efficiency but also other significant sleep outcomes, such as sleep architecture.

## 5. Conclusions

In accordance with our findings, an increased subjective sleep quality was associated with higher S-Klotho plasma levels in sedentary middle-aged adults. Moreover, TST, WASO, and SE were positively related to the S-Klotho plasma levels after controlling for confounders. Therefore, improving sleep quantity and quality could be considered an anti-aging therapeutic approach for the prevention, slowing, and even reversal of the physiological decline and degenerative pathologies that are certainly related to the aging process.

## Figures and Tables

**Table 1:** Descriptive characteristics.

Outcome	*N*	All		*N*	Men		*N*	Women	
Age (years)	74	53.66	(5.14)	35	54.39	(5.27)	39	53.01	(5.00)
Geographical origin of the population (*n*/%)	74			35			39		
Spain		74	(100.0)		35	(100.0)		39	(100.0)
Place of residence (*n*/%)	74			35			39		
Urban		63	(85.1)		30	(84.7)		33	(84.6)
Rural		11	(14.9)		5	(15.3)		6	(15.4)
Socio-professional category (*n*/%)	74			35			39		
Technicians and professional intellectual scientists		1	(1.35)		0	(0.00)		1	(2.56)
Technicians and associate professionals		3	(4.05)		1	(2.86)		2	(5.13)
Service and sales workers		4	(5.41)		0	(0.00)		4	(10.26)
Skilled agricultural, forestry and fishery workers		43	(58.11)		23	(65.71)		20	(51.28)
Unemployed		2	(2.70)		2	(5.71)		0	(0.00)
Elementary occupations		16	(21.62)		6	(17.14)		10	(25.64)
Others		5	(6.76)		3	(8.58)		2	(5.13)
S-Klotho plasma levels (pg/mL)	73	775.3	(363.7)	34	814.1	(452.2)	39	741.4	(265.6)
Antropometry and Body composition									
Height (cm)	74	167.8	(9.81)	35	175.8	(6.48)	39	160.7	(6.10) *
Weight (kg)	74	75.73	(14.98)	35	87.38	(10.95)	39	65.28	(9.32) *
Body mass index (kg/m^2^)	74	26.72	(3.76)	35	28.32	(3.61)	39	25.27	(3.31) *
Fat mass (%)	74	39.90	(9.06)	35	34.75	(7.99)	39	44.52	(7.36) *
Fat mass index (kg/m^2^)	74	10.75	(3.13)	35	10.03	(3.23)	39	11.39	(2.93)
Lean mass index (kg/m^2^)	74	15.21	(2.88)	35	17.49	(2.02)	39	13.17	(1.80) *
Sleep quantity and quality									
Objective sleep quantity and quality									
Total sleep time (min)	71	359.9	(48.85)	34	340.1	(47.72)	37	378.1	(42.88) *
Wake after sleep onset (min)	71	63.90	(27.44)	34	71.28	(32.70)	37	57.12	(19.63) *
Sleep efficiency (%)	71	85.01	(6.29)	34	82.89	(7.41)	37	86.96	(4.28) *
Subjective sleep quantity and quality									
Subjective sleep quality	67	1.13	(0.82)	31	0.84	(0.78)	36	1.39	(0.77) *
Sleep latency	67	1.07	(0.86)	31	1.03	(0.88)	36	1.11	(0.85)
Sleep duration	67	0.99	(0.77)	31	0.97	(0.66)	36	1.00	(0.86)
Habitual sleep efficiency	67	0.60	(0.95)	31	0.32	(0.75)	36	0.83	(1.06) *
Sleep disturbances	67	1.13	(0.42)	31	1.03	(0.41)	36	1.22	(0.42)
Use of sleeping medication	67	0.31	(0.76)	31	0.19	(0.60)	36	0.42	(0.87)
Daytime dysfunction	67	0.37	(0.55)	31	0.39	(0.50)	36	0.36	(0.59)
Global PSQI score	67	5.61	(3.47)	31	4.77	(3.15)	36	6.33	(3.62)

Data are presented as means (standard deviation). * Significant differences between sexes obtained from an independent sample *t*-Test (*p* < 0.05). S-Klotho = Secreted Klotho; PSQI = Pittsburgh Sleep Quality Index.

**Table 2:** Association of objective sleep quantity and quality with the secreted Klotho plasma levels (Model 0) adjusted by age (Model 1), total fat mass percentage (Model 2), fat mass index (Model 3), and lean mass index (Model 4).

Model	All			Men			Women		
	*β*	*R* ^2^	*p*	*β*	*R* ^2^	*p*	*β*	*R* ^2^	*p*
Total sleep time									
Model 0	−0.111	0.012	0.362	−0.094	0.009	0.601	−0.065	0.004	0.702
Model 1	−0.057	0.482	**<0.001**	−0.033	0.657	**<0.001**	0.102	0.419	**<0.001**
Model 2	0.031	0.106	**0.023**	−0.029	0.060	0.393	0.065	0.251	**0.007**
Model 3	−0.094	0.015	0.597	−0.106	0.010	0.857	−0.038	0.043	0.476
Model 4	0.137	0.356	**<0.001**	−0.069	0.722	**<0.001**	0.162	0.549	**<0.001**
Wake after sleep onset									
Model 0	0.100	0.010	0.409	0.071	0.005	0.693	0.098	0.010	0.566
Model 1	0.108	0.491	**<0.001**	−0.028	0.656	**<0.001**	0.156	0.433	**<0.001**
Model 2	−0.012	0.106	**0.024**	−0.007	0.060	0.398	0.036	0.248	**0.008**
Model 3	0.083	0.014	0.633	0.082	0.006	0.912	0.083	0.048	0.433
Model 4	−0.054	0.343	**<0.001**	0.002	0.717	**<0.001**	0.036	0.526	**<0.001**
Sleep efficiency									
Model 0	−0.099	0.010	0.413	−0.062	0.004	0.732	−0.103	0.011	0.544
Model 1	−0.107	0.491	**<0.001**	0.013	0.656	**<0.001**	−0.108	0.421	**<0.001**
Model 2	0.035	0.106	**0.023**	0.020	0.060	0.396	−0.011	0.247	**0.008**
Model 3	−0.081	0.013	0.644	−0.073	0.005	0.932	−0.080	0.048	0.436
Model 4	0.093	0.348	**<0.001**	−0.004	0.717	**<0.001**	0.000	0.525	**<0.001**

*β* (standardized regression coefficient), *R^2^*, and *p*-value of simple and multiple-regression analysis. Significant *p* values (<0.05) are in bold.

**Table 3:** Association of subjective sleep quantity and quality (components and global PSQI score) with the secreted Klotho plasma levels (Model 0) adjusted by age (Model 1), total fat mass percentage (Model 2), fat mass index (Model 3), and lean mass index (Model 4).

Model	All			Men			Women		
	*β*	*R* ^2^	*p*	*β*	*R* ^2^	*p*	*β*	*R* ^2^	*p*
Subjective sleep quality									
Model 0	−0.368	0.135	**0.002**	−0.351	0.123	0.057	−0.412	0.170	0.013
Model 1	−0.275	0.573	**<0.001**	−0.146	0.633	**<0.001**	−0.318	0.599	**<0.001**
Model 2	−0.250	0.191	**0.001**	−0.302	0.166	0.087	−0.188	0.399	**<0.001**
Model 3	−0.349	0.139	**0.009**	−0.354	0.124	0.168	−0.334	0.224	**0.015**
Model 4	−0.131	0.332	**<0.001**	−0.063	0.696	**<0.001**	−0.195	0.535	**<0.001**
Sleep latency									
Model 0	−0.519	0.269	**<0.001**	−0.565	0.319	0.001	−0.483	0.234	0.003
Model 1	−0.350	0.611	**<0.001**	−0.354	0.726	**<0.001**	−0.319	0.594	**<0.001**
Model 2	−0.451	0.331	**<0.001**	−0.528	0.336	**0.004**	−0.335	0.473	**<0.001**
Model 3	−0.505	0.275	**<0.001**	−0.572	0.322	**0.005**	−0.432	0.304	**0.003**
Model 4	−0.384	0.453	**<0.001**	−0.196	0.722	**<0.001**	−0.295	0.581	**<0.001**
Sleep duration									
Model 0	−0.140	0.020	0.261	−0.153	0.023	0.420	−0.147	0.021	0.394
Model 1	−0.101	0.509	**<0.001**	−0.091	0.622	**<0.001**	−0.113	0.512	**<0.001**
Model 2	−0.119	0.156	**0.005**	−0.107	0.091	0.277	−0.163	0.397	**<0.001**
Model 3	−0.136	0.046	0.228	−0.152	0.023	0.726	−0.162	0.150	0.068
Model 4	−0.095	0.328	**<0.001**	0.015	0.693	**<0.001**	−0.128	0.518	**<0.001**
Habitual sleep efficiency									
Model 0	−0.277	0.077	**0.024**	−0.443	0.197	**0.014**	−0.140	0.020	0.414
Model 1	−0.209	0.542	**<0.001**	−0.252	0.673	**<0.001**	−0.087	0.507	**<0.001**
Model 2	−0.215	0.186	**0.002**	−0.424	0.258	**0.018**	−0.123	0.385	**<0.001**
Model 3	−0.282	0.107	**0.028**	−0.451	0.202	**0.048**	−0.177	0.155	0.062
Model 4	−0.065	0.322	**<0.001**	−0.064	0.696	**<0.001**	0.010	0.502	**<0.001**
Sleep disturbances									
Model 0	−0.407	0.165	**0.001**	−0.445	0.198	**0.014**	−0.376	0.141	**0.024**
Model 1	−0.125	0.511	**<0.001**	−0.070	0.617	**<0.001**	−0.057	0.502	**<0.001**
Model 2	−0.335	0.247	**<0.001**	−0.408	0.241	**0.024**	−0.301	0.459	**<0.001**
Model 3	−0.400	0.187	**0.001**	−0.445	0.198	0.051	−0.396	0.280	**0.004**
Model 4	−0.207	0.354	**<0.001**	−0.131	0.707	**<0.001**	−0.129	0.516	**<0.001**
Use of sleeping medication									
Model 0	−0.150	0.023	0.229	−0.378	0.143	**0.039**	0.062	0.004	0.720
Model 1	−0.158	0.523	**<0.001**	−0.228	0.663	**<0.001**	−0.010	0.500	**<0.001**
Model 2	−0.088	0.149	**0.006**	−0.323	0.175	0.074	0.069	0.375	**<0.001**
Model 3	−0.143	0.048	0.214	−0.378	0.143	0.125	0.051	0.127	0.107
Model 4	0.009	0.319	**<0.001**	0.103	0.700	**<0.001**	0.135	0.520	**<0.001**
Daytime dysfunction									
Model 0	−0.211	0.045	0.089	−0.335	0.112	0.071	−0.102	0.010	0.555
Model 1	−0.137	0.517	**<0.001**	−0.234	0.668	**<0.001**	−0.044	0.502	**<0.001**
Model 2	−0.188	0.177	**0.002**	−0.381	0.222	**0.034**	0.074	0.375	**<0.001**
Model 3	−0.206	0.070	0.103	−0.353	0.121	0.176	−0.025	0.125	0.111
Model 4	−0.161	0.344	**<0.001**	−0.169	0.720	**<0.001**	0.007	0.502	**<0.001**
Global PSQI score									
Model 0	−0.438	0.192	**<0.001**	−0.563	0.317	**0.001**	−0.323	0.104	0.055
Model 1	−0.304	0.587	**<0.001**	−0.323	0.704	**<0.001**	−0.209	0.542	**<0.001**
Model 2	−0.355	0.255	**<0.001**	−0.525	0.339	**0.004**	−0.197	0.407	**<0.001**
Model 3	−0.423	0.204	**0.001**	−0.563	0.317	**0.006**	−0.292	0.208	**0.021**
Model 4	−0.236	0.364	**<0.001**	−0.131	0.704	**<0.001**	−0.118	0.515	**<0.001**

***β*** (standardized regression coefficient), *R^2^*, and *p*-value of simple and multiple-regression analysis. Significant *p* values (<0.05) are in bold. PSQI = Pittsburgh Sleep Quality Index.
